# Attention-Based Fusion of Ultrashort Voice Utterances and Depth Videos for Multimodal Person Identification

**DOI:** 10.3390/s23135890

**Published:** 2023-06-25

**Authors:** Abderrazzaq Moufidi, David Rousseau, Pejman Rasti

**Affiliations:** 1 Centre d’Études et de Recherche pour l’Aide à la Décision (CERADE), ESAIP, 18 Rue du 8 Mai 1945, 49124 Saint-Barthélemy-d’Anjou, France; abderrazzaq.moufidi@univ-angers.fr; 2 Laboratoire Angevin de Recherche en Ingénierie des Systèmes (LARIS), UMR INRAe-IRHS, Université d’Angers, 62 Avenue Notre Dame du Lac, 49000 Angers, France; david.rousseau@univ-angers.fr

**Keywords:** depth images, lip identification, speaker identification, late fusion, multimodality, spatiotemporal

## Abstract

Multimodal deep learning, in the context of biometrics, encounters significant challenges due to the dependence on long speech utterances and RGB images, which are often impractical in certain situations. This paper presents a novel solution addressing these issues by leveraging ultrashort voice utterances and depth videos of the lip for person identification. The proposed method utilizes an amalgamation of residual neural networks to encode depth videos and a Time Delay Neural Network architecture to encode voice signals. In an effort to fuse information from these different modalities, we integrate self-attention and engineer a noise-resistant model that effectively manages diverse types of noise. Through rigorous testing on a benchmark dataset, our approach exhibits superior performance over existing methods, resulting in an average improvement of 10%. This method is notably efficient for scenarios where extended utterances and RGB images are unfeasible or unattainable. Furthermore, its potential extends to various multimodal applications beyond just person identification.

## 1. Introduction

Multimodal deep learning has emerged as a powerful approach for various tasks by combining information from different modalities, exploiting their complementary nature, and enhancing their overall performance [1,2,3,4]. In the realm of speaker recognition, incorporating multiple features, such as lip movements, depth images, and voice, can lead to improved accuracy and robustness in applications such as security systems, access control, and surveillance [2,5,6,7,8,9].

Utilizing a combination of physiological and behavioral features, including the face, iris, fingerprints [10], voice [11], and lip movements, can benefit person identification [2,5,6]. While each feature offers unique advantages and limitations, integrating them using multimodal deep learning methods can lead to more reliable identification systems [2,3]. Among these features, lip movements serve as a vital behavioral feature for speaker recognition encompassing both physiological (static) and behavioral (temporal) aspects [6].

Traditional methods, such as RGB images and videos, have been commonly used for lip verification [2,5,6,12]. However, they are susceptible to issues such as varying lighting conditions, pose variations, and occlusions. In contrast, depth images offer several advantages over traditional RGB images. They are more robust against attempts to deceive recognition systems using photographs and exhibit strong invariance to lighting conditions, which can adversely affect RGB images in challenging environments [13].

Voice-based identification is another important aspect [11]. While it has been widely studied [11], acquiring long and clear voice samples can be challenging [14,15,16], especially in real-world scenarios such as crowded places [17]. This necessitates the use of ultrashort voice utterances (less than 1 s) for identification. In this paper, we propose a novel multimodal deep learning approach for person identification using depth images and ultrashort voice utterances, addressing the limitations of existing methods on each modality separately.

One of the challenges in multimodal deep learning is effectively fusing information from different modalities [3]. Existing fusion techniques, such as early, late, and hybrid fusion, have their inherent limitations, often resulting in suboptimal integration of multimodal information [1,18]. Our proposed attention-based fusion model effectively addresses these limitations, enabling the model to better adapt to the varying degrees of informativeness in different modalities and achieve improved performance.

Despite the significant strides made in multimodal deep learning, a clear research gap persists when applied to speaker recognition. The existing methods rely heavily on long speech utterances and RGB images for identification. This dependence often leads to impractical and inconsistent outcomes, especially in scenarios where obtaining extended utterances or ideal lighting conditions for RGB images is challenging. Additionally, these methods face difficulties with robustly handling various types of noise, which are typical in real-world situations. Our study directly addresses this gap by developing a novel approach that utilizes ultrashort voice utterances and depth videos of the lips, ensuring practicality and improved accuracy even in less than ideal conditions.

In this study, we introduce a fresh perspective on the application of multimodal deep learning for speaker recognition. The crux of our innovation is a distinctive encoding model that processes time series depth images of the lips and ultrashort voice utterances, demonstrating superior performance on benchmark datasets despite the limited information available. This is paired with an attention-based model that enhances identification accuracy by effectively fusing multimodal data and focusing on the most informative regions. Therefore, the main contributions and novelty of our work are listed as follows:

- We introduce a new encoding model for time series depth images of the lips and ultrashort voice utterances, which leverages convolutional networks to capture both spatial and temporal features. Despite the challenges posed by the limited information available in short utterances and depth images, our encoding model demonstrates superior performance on benchmark datasets.
- We propose an attention-based model for the fusion of multimodal data that effectively combines information from different modalities, such as the lips and ultrashort voice utterances, and depth images. The attention mechanism allows the model to concentrate on the most informative regions of the input data, enhancing the accuracy of person identification.
- Our deep learning model is robust to various noises, such as ambient noise and background music, thanks to the depth modality, which can significantly impact identification accuracy in real-world scenarios. In contrast to many state-of-the-art methods, our model effectively handles different types of noise and achieves a superior performance.
- We present a comprehensive evaluation of our proposed approach on benchmark datasets, demonstrating its effectiveness in challenging scenarios with ultrashort voice utterances (less than 1 s) and depth videos. This showcases the potential of our method for real-world applications, such as security systems and access control.

## 2. Related Works

In the field of biometric identification, comprehensive studies have been conducted on unimodal systems [6,11,12]. This includes speaker identification through audio input [16,19,20,21,22,23] as well as lip and facial identification via visual and behavioral factors [5,6,7,12,13]. However, each of these modes has potential drawbacks and limitations based on the conditions under which recordings are made. To address these challenges, multimodal biometric identification [2,3,24] brings together several modalities. This approach utilizes the synergies among different modalities to provide a resilient solution to issues such as a a noisy environment or poor lighting conditions. Consequently, our research is primarily centered around multimodal individual identification using a weighted late fusion of lip depth videos and associated vocal patterns. This area has been largely studied with the use of RGB lip-audio [2,6] or RGB-D face-audio [3] fusion. However, the efficacy of fusing lip depth and audio modalities under real-world conditions, such as short utterances, inadequate lighting, or a noisy atmosphere, has not been thoroughly examined.

The lips play a major role in voice production. They modulate the voice coming from the vocal cord vibrations; in other words, they are highly correlated with human speech [2,6]. Therefore, optimal fusion of lip movement and audio can represent a major improvement. Lip biometric recognition can be further categorized into physiological and behavioral characteristics, providing both static and temporal attributes that are specific to a person [6]. Various techniques have been employed in the literature to extract the physiological and morphological features of the lips.

For the geometric lip recognition task, [12] introduced a Probabilistic Neural Network (PNN), beginning with the detection of lip landmarks from RGB facial images. Subsequently, geometric features were extracted based on lip contours from a still image, which were then input into a PNN for classification purposes. The referenced studies underscore the significance of the lips’ spatial appearance as a crucial attribute for encoding person identity. Based on this premise, it is imperative for our person identification system to incorporate feature extraction from the visual modality which, in our case, is depth.

In contrast to physiological features, biometric characteristics can also be identified through lip movements [2,6]. A novel feature extraction technique for lip motion was proposed by [5], which involved estimating the optical flow of the mouth region in RGB videos. This was achieved by projecting the speaker’s video (3D spatiotemporal data) onto 2D planes (XT and YT planes). The resulting visual features were found to enhance identification accuracy on the XM2VTS database [25], particularly when combined with audio features. This demonstrates the potential for improved performance by fusing different modalities in speaker identification tasks and the contribution that can be added by encoding lip movements (temporal information) with our identification system.

The combination of audio and visual information has been shown to positively impact biometric recognition [2,3]. These two modalities can complement one another with audio-only and depth identification being resilient against static images, such as photographs. In a study by [2], the dynamic visual RGB information from the mouth region was combined with voice data. The authors employed late fusion, wherein the characteristics of each modality were extracted separately and the feature vectors were vertically concatenated for classification. In another approach, Schönherr [3] proposed a weighted multimodal identification method, which fused different modalities (RGB and Depth as well as Face and Audio) at the score level to achieve robust biometric recognition. This method demonstrated that the presence of noise in audio or visual modalities did not negatively impact the results compared to an unweighted combination. Drawing from these findings, our identification system is designed to harness the complementary information sourced from two distinct modalities (audio and depth). By utilizing a weighted late fusion approach, we aim to offset the shortcomings inherent in each modality while simultaneously capitalizing on their respective strengths.

Feature extraction from lip depth videos, which is a crucial part of our task, poses intricate challenges [1]. The common approach involves the use of 3D Convolutional Neural Networks (CNNs), known for their ability to extract temporal and spatial correlations with diverse granularity. However, 3D CNNs require an extensive array of parameters and a lengthy training period, creating significant obstacles [26]. In response to these issues, we leveraged a unique strategy from our previous work [26], where we developed a technique to project 4D (3D + times) Magnetic Resonance Imaging (MRI) data (XYZT) onto separate (XYZ) and (XYT) planes for individual processing. This innovative approach effectively addresses the difficulties associated with 3D CNNs, and critically, it was proven to retain its efficiency when applied to our current work: extracting physiological and behavioral features from lip depth videos (2D + times) in our person identification system. This approach, combined with feeding these components separately into a CNN model, could potentially alleviate the computational challenges linked with higher-dimensional analysis while retaining the capability to detect meaningful patterns and features. This novel approach is positioned to enhance the efficacy and efficiency of spatiotemporal data analysis, creating new potential for research and applications in various domains. For audio feature extraction, we chose the x-vectors architecture presented in [20], which yields a signature vector of a given human speech pattern. For the fusion stage, we developed a weighted late fusion method based on the attention mechanism.

## 3. Methodology

Drawing from the literature that explores various sources of information, such as those from diverse modalities or spatiotemporal data, our primary goal is to recognize individuals using depth visual information, dynamic lip movements, and brief audio utterances lasting less than one second. To efficiently extract valuable information from our chosen modalities, namely audio and depth, we decided to employ a late fusion strategy. This approach entails the feeding of each modality into a network specifically designed to handle the structure of the respective information. The resulting feature vectors from both systems are then combined to make a decision regarding the speaker’s identity. In the following sections, we outline the CNN employed to extract features from each modality based on the existing literature.

When transitioning from one modality to another, we must ensure that the network architecture is designed to optimally process the specific data structure. This allows us to maximize the accuracy and efficiency of the speaker identification process. By adopting a late fusion strategy, we can effectively combine the strengths of each individual modality to improve the overall performance.

As we delve further into this topic, we discuss the details of the CNN used for feature extraction in each modality. By drawing upon the work conducted in the literature, we aim to create a robust and efficient system that can accurately identify speakers based on their depth visual information, dynamic lip movements, and short audio utterances.

### 3.1. Voice Speaker Identification

To identify a speaker from brief speech segments, we employed pretrained models from SpeechBrain [27] and Hugging Face [28] that were initially trained on the TIMIT [29] and VoxCeleb datasets [17]. We evaluated various models, such as SincNet [14], x-vectors [20], and ECAPA-TDNN [19], to find the most appropriate architecture for our KinectsDigits [3] and TCD-TIMIT [30] data. Although the article does not discuss the performance differences between these models, x-vectors [20] were chosen due to their reduced overfitting issues. Furthermore, for the VoxCeleb2 dataset [17], we fine-tuned the x-vectors [20] architecture using ultrashort utterances (<1 s) after initially conducting transfer learning with x-vectors [20] pretrained on both the VoxCeleb1 and VoxCeleb2 [17,28] datasets.

All audio samples from the three benchmark datasets were either originally sampled or resampled at a 16 kHz frequency. Before inputting the audio signal into the x-vectors [20] architecture, an MFCC [31] transform with a 25 ms frame length and a 10 ms overlap was applied. The cepstrum coefficients were mean normalized across the word duration, instead of the 3 s mentioned in the reference article [20], resulting in a feature vector $X_{a}\in R^{512}$. For training x-vectors [20] on ultrashort utterances from VoxCeleb2 [17], a batch size of 32 was used, along with the ADAMAX optimizer and a learning rate of $5\times 10^{-3}$.

### 3.2. Lip Identification

In the realm of voice production, a robust correlation exists between lip movement and the ensuing audio, as the lips serve to modulate the vibrations of the vocal cords, particularly within the lower-frequency portions of the audio spectrum. The literature has demonstrated that these low-frequency components encompass both dynamic and visual biometric elements [6]. Guided by this knowledge, we devised a novel spatiotemporal architecture, which aims to extract not only spatial features but also temporal characteristics from the used data.

**Figure 1:**
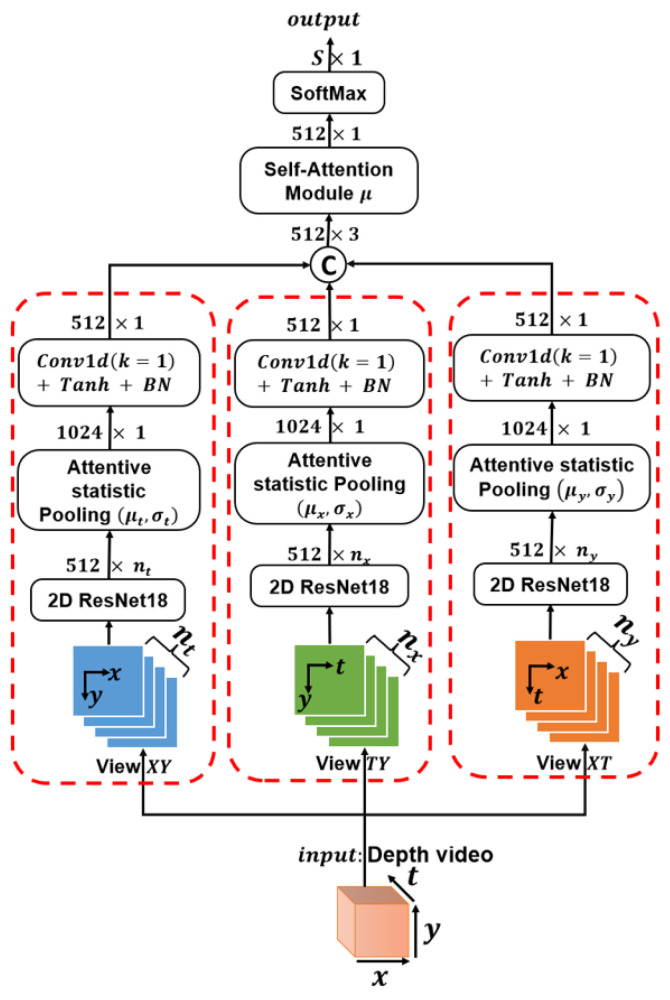
Multiview Video CNN architecture used on lip depth videos (The red dashed line represents the extraction of the features vector from the view projection of the video).

As mentioned in the related works section, 3D CNN is a popular architecture for feature extraction from videos. However, this architecture demands a significant number of parameters and more time compared to 2D CNN. To address this challenge, we propose the projection of 3D data (2D space + time) onto all possible 2D combinations, namely (X,Y),(X,T),(T,Y). Subsequently, each 2D combination undergoes mean normalization as a distance calibration and is fed into a ResNet18 neural network pretrained on ImageNet [32], as delineated in Figure 1.

For every view, (X,Y), (X,T), and (T,Y), the network produces a two-dimensional output matrix, $Y_{in}\in R^{512\times n}$, where *n* represents the quantity of frames along the absent dimension. The first pair of static parameters across this missing third dimension is gleaned through an attentive statistic pooling technique [19].

The input $Y_{in}$ of this module passes by a Tanh activation and 1D-Conv (same input–output channels) layer to calibrate the channel weight (first line in Equation (1)), the output score $Y_{in1}\in R^{512\times n}$ is normalized across the missing dimension by using the softmax function, and the generated weights $W_{c,i}$ (c=1,...,512 is the channel index and i=1,...,n is the frame index) are then used to compute the weighted mean $\mu_{c}$ and the standard deviation $\sigma_{c}$ across the third axis.

This module describes importance to a frame (space or time) contingent on the ability to augment feature invariance, ultimately yielding a vector $Y_{out}\in R^{1024}$ for each view {(X,Y),(X,T),(T,Y)}.

(1) $$\begin{array}{cc} Y_{in1} & =\mathrm{Conv}\left(k=1\right)\left(\mathrm{Tanh}\left(Y_{in}\right)\right)\in R^{512\times n},\\ W_{c,i} & =\frac{\exp\left(Y_{in1}\left(c,i\right)\right)}{\sum_{i=1}^{n}\exp\left(Y_{in1}\left(c,i\right)\right)},\\ \mu_{c} & =\sum_{i=1}^{n}W_{c,i}Y_{in1}\left(c,i\right),\\ \sigma_{c} & =\sqrt{\sum_{i=1}^{n}W_{c,i}{\left(Y_{in1}\left(c,i\right)-\mu_{c}\right)}^{2}},\\ Y_{\mathrm{out}\;} & ={\left(\mu_{1},\;\ldots ,\;\mu_{512},\;\sigma_{1},\;\ldots ,\;\sigma_{512}\right)}^{T}\in R^{1024} \end{array}$$

To derive a holistic understanding of the video, the resultant vectors from the attentive static pooling operation $Y_{out_{\left(X,Y\right)}}$, $Y_{out_{\left(T,Y\right)}}$, and $Y_{out_{\left(X,T\right)}}$ are projected onto the space $R^{512}$ through a 1D convolution employing a kernel size of 1, followed by batch normalization and the implementation of a Tanh activation function. The projected vectors are concatenated and averaged by utilizing the self-attention module.

We introduce a self-attention module built upon the foundation of the attentive static pooling concept with the distinct purpose of optimizing both the channel and view characteristics. The vector $Z_{in}\in R^{512\times 3}$, which arises from the concatenation of the three views, is subjected to Tanh activation and a 1D-Conv layer with the same input–output channel, 512, enabling channel weight calibration. Following this, the transposed output vector $Z_{in1}$ is passed through a Tanh activation and a 1D-Conv layer with the same input–output channel, 3, as illustrated by the second equation in Equation (2). This final step allows for the calibration of the weights of the views.

The output vector $Z_{in2}$ is normalized across the views axis using the softmax function, and the generated scores $W_{c,i}^{\prime }$ (c=1,...,512 is the channel index and i=1,2,3 is the view index) are then used to compute the weighted mean $\mu_{c}^{\prime }$ across the views axis. Therefore, the generated vector $Z_{out}\in R^{512}$ captures the comprehensive spatiotemporal information from the video and shares an identical dimension with the audio feature vector

(2) $$\begin{array}{cc} Z_{in1} & =\mathrm{Conv}\left(k=1\right)\left(\mathrm{Tanh}\left(Z_{in}\right)\right)\in R^{512\times 3},\\ Z_{in2}^{T} & =\mathrm{Conv}\left(k=1\right)\left(\mathrm{Tanh}\left(Z_{in1}^{T}\right)\right)\in R^{3\times 512},\\ W_{c,i}^{\prime } & =\frac{\exp\left(Z_{in2}\left(c,i\right)\right)}{\sum_{i=1}^{3}\exp\left(Z_{in2}\left(c,i\right)\right)},\\ \mu_{c}^{\prime } & =\sum_{i=1}^{3}W_{c,i}^{\prime }Z_{in2}\left(c,i\right),\\ Z_{\mathrm{out}\;} & ={\left(\mu_{1}^{\prime },\;\ldots ,\;\mu_{512}^{\prime }\right)}^{T}\in R^{512}. \end{array}$$

To train this depth view fusion system, a batch size of 32 was used, along with the ADAMAX optimizer and a learning rate of $10^{-2}$.

### 3.3. Fusion of Depth and Audio Modalities

**Figure 2:**
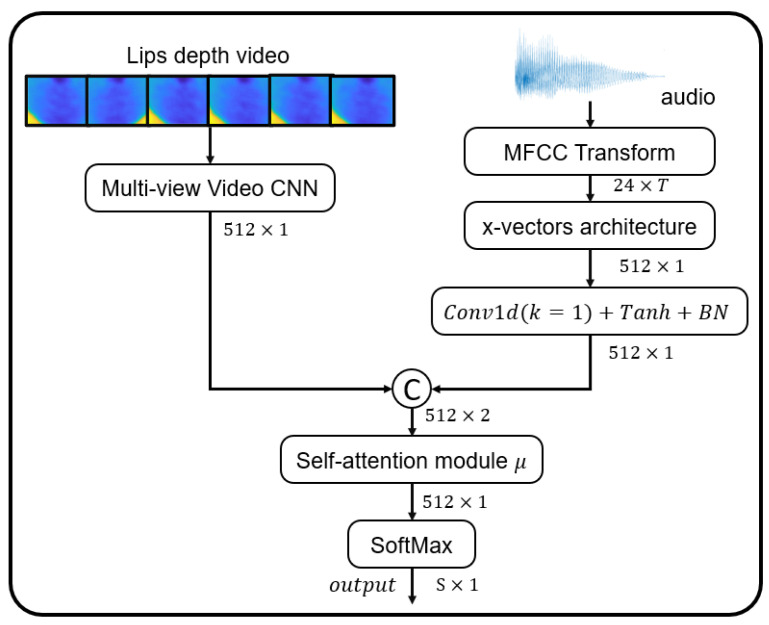
Architecture of late fusion of the two modalities (audio and depth video).

In our proposed method, we employ a late fusion architecture, as illustrated in Figure 2. This architecture utilizes distinct networks designed to handle the unique information present in each modality to ensure each modality’s specific characteristics are appropriately processed. For the audio component, we apply a 1D convolution with a kernel size of 1 to the x-vector outputs. This procedure is further enhanced with batch normalization and a Tanh activation function. For the depth video modality, we utilize a multiview CNN (as shown in Figure 1) on the depth video to extract a feature vector $X_{d}\in R^{512}$, encapsulating both visual and dynamic lip movement information.

Following the individual processing of these modalities, the resultant vectors are routed through our self-attention module (illustrated in Figure 2). This module calculates the weighted sum of the two vectors as per Equation (2). Our approach is informed by the insights of [3], underscoring the advantages of using weighted fusion. By utilizing this strategy, we minimize the potential redundancy or ambiguity associated with each modality, reducing contradictions and significantly boosting the overall performance of our model.

## 4. Experimental Results and Discussion

### 4.1. Data Collection

To evaluate our proposed methodology, we used three well-known benchmark datasets: KinectsDigits [3], VoxCeleb2 [17], and TCD-TIMIT [30].

KinectsDigits [3] is a valuable resource for multimodal speaker recognition research. The dataset, captured using Microsoft’s Kinect sensor, consists of RGB, depth, and audio modalities. It features recordings of individuals articulating digits from zero to nine under various environmental conditions. We only kept one situation to prevent repeated words. The videos had a resolution of 104×80 pixels, a frame rate of 30 fps, and a voice signal sampling rate of 16 kHz.

**Figure 3:**
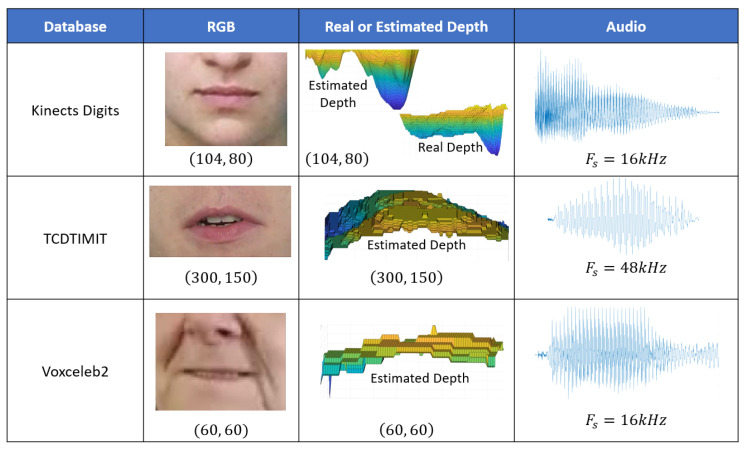
General Information on the three benchmarks used during the study (Fs represents the frequency sampling before re-sampling to 16 kHz; the audio curves are the full utterance or a part of a word spoken by a speaker in each dataset).

VoxCeleb2 [17] is an essential resource for speaker recognition research, containing RGB and audio modalities from a diverse range of speakers. The dataset features over a million utterances from thousands of speakers with various accents, languages, and speech contents. Videos in VoxCeleb2 have a 25 fps and a low resolution of 224×224, while audio signals are sampled at 16 kHz. As we used a very low resolution, we predicted that the depth estimation network [33] would give less details about the lip region, as shown on Figure 3; therefore, we only selected 1000 random speakers from this dataset.

TCD-TIMIT [30] is a valuable resource for multimodal speech recognition research, comprising continuous speech in both RGB and audio modalities. The dataset features individuals delivering sentences from the TIMIT corpus. Videos in the TCD-TIMIT database have a resolution of 1920×1080 pixels, a frame rate of 29 fps, and a voice signal sampling rate of 48 kHz. Only audio signals were re-sampled to 16 kHz.

While KinectsDigits [3] includes RGB, depth, and audio modalities, VoxCeleb2 [17] and TCD-TIMIT [30] only provide RGB and audio data. Our experimental approach focused on the depth information from the speakers’ lips, present in KinectsDigits [3] but not in the other two datasets. To ensure consistency across all datasets, we applied a lip detection and depth estimation technique to the RGB videos of the three databases, obtaining the same modalities for the three benchmarks. The following subsections outline our lip detection and depth estimation strategies.

#### Depth Estimation

The primary aim of this research was to demonstrate the synergistic value of depth and audio data for multimodal person identification. However, most publicly accessible datasets suitable for this purpose only include RGB video and audio modalities. To address this limitation, we utilize a pretrained face depth estimation network (employing an encoder–decoder architecture) [33] to generate depth maps from full RGB facial images. This method, trained on an extensive synthetic face dataset with varying head poses, expressions, backgrounds, and image resolutions, outperforms state-of-the-art models on real face depth datasets, such as Pandora [34], Eurecom Kinect Face [35], and the Biwi Kinect Head Pose dataset [36]. Consequently, this model can produce a depth modality for RGB faces in datasets.

The depth estimation network was independently applied to each facial frame within a given video for Voxceleb2 and TCD-TIMIT. For the KinectsDigits dataset, we retrained the depth estimation network on the Pandora [34] lip regions to enable a fair comparison between the estimated and actual depth in the person identification task. Although the resulting depth estimation may be considered low resolution due to some missing depth information, it remains a valuable data source for our analysis, as evidenced in the results section. The Figure 3 presents a sample of real or estimated depth mouth crop frames for the three benchmark datasets.

***Lip extraction***

In our approach to data preparation, we employed an existing method for lip detection and applied it to two supplementary datasets, namely VoxCeleb2 [17] and TCD-TIMIT [30]. The aim was to accurately identify and extract the lip region of speakers in RGB videos for further analysis. To accomplish this, we utilized Google’s Mediapipe tool. By leveraging the power of Mediapipe, we were able to identify the key landmarks associated with the mouth region. This allowed us to isolate the lower part of the face on the depth video and focus our attention on the lips.

***Word segmentation***

In our investigation of the ultrashort utterance problem, we segmented words from the audio in both the VoxCeleb2 and TCD-TIMIT datasets (while the KinectsDigits audio only contained digit words from zero to nine). We removed duplicate instances of the same word for each speaker to concentrate on the text-independent speaker identification task. Subsequently, we interpolated the resulting timestamps on the video to extract the lip video section corresponding to the spoken word. We utilized models from the Kaldi toolkit for word segmentation, which are available for various languages. It is worth noting that, for some words, we obtained a single frame, which the spatiotemporal network processed as 3D data, just like longer videos.

Table 1 presents pertinent information regarding each dataset and its usage, with an 80%/20% training/testing split. Remarkably, up to 95% of the data has a duration of less than 1 s, demonstrating that the task was focused solely on short utterance identification.

### 4.2. Results

This section addresses the experimental results of the proposed architecture in Figure 2 and its components applied on the three datasets. We investigate the performance of our spatiotemporal fusion system, the importance of adding depth information to audio, and the complementary information added by each modality. To understand how the fusion of modalities can improve the performance of the system, we first examine each individual modality and its identification abilities.

We begin our discussion by exploring the impact of depth video encoding. The fusion of all three views, as depicted in Figure 1, led to a noticeable improvement of approximately 2% in performance. This improvement validates the significant advantage of integrating both spatial and temporal information into a single modality. Furthermore, it is noteworthy to emphasize the critical role that the self-attention module played in these results. By finely balancing the spatial and temporal information, this module effectively amplified the overall performance of the speaker identification process.

To provide additional insight, we dove deeper into the contribution of each view towards person identification based on the depth video of their lip. To achieve this, we dissected the identification performance for each 2D view of the video using the architecture illustrated in Figure 1. The ensuing findings, presented in Table 2, allow for a thorough analysis of the various views.

The analysis unveils that the (XY) view, which encapsulates the spatial information from the video, has a substantial edge over the other views. Its performance improvement is quite significant: 5% for the KinectsDigits dataset trained on the real or estimated depth, 9.27% for TCD-TIMIT, and 8.08% for VoxCeleb2. Following the (XY) view, the horizontal view (XT), which covers the horizontal movements of the lip, showed remarkable results on the real depth KinectsDigits dataset [3]. In fact, it surpassed the (TY) view by 8.33%, indicating the importance of temporal dynamics. Furthermore, the performance improvement from depth estimated video reveals an interesting prospect of considering the valuable information provided by the estimated depth.

Diving into another critical aspect of our analysis, the audio component of videos, we discovered its pronounced significance in the sphere of speaker identification tasks. We used the x-vectors architecture, a powerful method for dealing with audio data. However, our results, as depicted in Table 3, highlight a considerable drop in performance, primarily attributed to the short duration of the voice samples. This brevity tended to trigger substantial overfitting, depending on the specificities of the dataset.

We found that the audio-based identification performed well in controlled recording environments, such as those present in the KinectsDigits [3] and TCD-TIMIT [30] datasets, where the identification accuracy surged beyond 75%. Nonetheless, in scenarios that are more reflective of real-world conditions, as is the case with the VoxCeleb2 [17] dataset, the accuracy nosedived to nearly half of its initial value. Such a drastic dip in accuracy raises concerns about the reliability of the system for accurate speaker recognition in practical applications.

The combination of different modalities can, however, offer a solution to this problem. Integrating various modalities not only harnesses their individual strengths but also supplements the limitations of each modality. Particularly, an effective fusion of audio and depth or estimated depth data can create a more robust and efficient system. Our results support this claim, as shown in Table 3, where we observed a marked enhancement in performance when both modalities were utilized in unison.

Upon fusing the modalities, the performance of audio identification shot up by 20%, while the real or estimated depth spatiotemporal encoding registered an average boost of 1.5%. These findings validate the significant potential of integrating the strengths of each modality. The result is a more potent speaker identification system that is capable of adjusting to diverse conditions and tackling the inherent challenges associated with short utterances and noisy recording environments effectively.

### 4.3. Discussion

These results not only emphasize the significant contribution of depth information to the overall performance, but they also highlight the valuable supplementary information provided by the audio component. By effectively combining these two modalities, it is possible to develop more advanced and reliable systems that capitalize on the strengths and complementary nature of both audio and depth information. Building on this observation, addressing the issue of speaker identification reliability requires the consideration of both audio and visual modalities. Fusing the information from these modalities results in a more robust and effective system that can overcome limitations posed by short utterances and variable recording environments. This multimodal approach significantly enhances the performance of speaker identification systems, ensuring their effectiveness in various real-world applications.

In speaker identification tasks, our spatiotemporal fusion method (illustrated in Figure 1) is an efficient way to process depth videos while deliberately avoiding the use of resource-intensive 3D CNNs. The logic behind steering clear of 3D CNNs is based on their inherent drawbacks, such as the high computational demand and memory requirements that can impede their use in real-time applications or on devices with limited resources. Furthermore, the greater number of parameters in 3D CNNs has potential implications for longer training times and increased susceptibility to overfitting, especially when working with datasets of limited size. In contrast, our chosen approach, which relies on a more efficient spatiotemporal fusion method, has the advantage of pulling out distinctive features from real or estimated depth videos, thus ensuring a comparably strong performance in speaker identification tasks without facing the challenges tied to the use of 3D CNNs, such as a high computational complexity and prolonged training periods.

This spatiotemporal fusion architecture consists of multiple 2D views, and an investigation into the contribution of each view is required. For the real depth data, specifically the KinectsDigits [3] dataset, the horizontal view of the mouth (XT) outperformed the vertical view (TY). This could be attributed to the mouth’s greater horizontal length, which enhances discriminant dynamic features. However, the (TY) view exhibited more pronounced and rapid overfitting, suggesting its susceptibility to overfitting in this context. It is crucial to select an appropriate mouth crop resolution, as the speaker identification performance can be significantly impacted by the resolution or depth estimation, as seen in the VoxCeleb [17] dataset.

Considering the spatial view (XY), it outperformed other fusions for both the estimated and real depths. This could be due to visual features conveying the 3D shape of the human face, while dynamic features only represent low frequencies of voice and behavior. The limited context for temporal features in short utterances might also contribute to this disparity. As the depth resolution increases, the gap between the (XY) view and other views grows larger, suggesting the increasing importance of spatial information for distinguishing speakers with improved resolution, leading to enhanced discriminative power for the (XY) view.

Another key element of our discussion is the analysis of errors that occur during the speaker identification process. Several noteworthy patterns emerged after examining the errors made by our proposed model. The primary source of error, especially for the audio modality, appears to be ultrashort utterances. Shorter utterances provide a limited temporal context, and the brevity of these utterances can lead to the omission of certain speaker-specific cues, thus causing the model to underperform in these cases. This issue is compounded in the VoxCeleb2 dataset, where the utterances are notably brief and the recording conditions are significantly more varied than in the other datasets, thus challenging the model’s performance.

Additionally, another observed pattern is the decrease in identification accuracy as the environmental noise increases, particularly with the VoxCeleb2 dataset. This dataset features recordings in diverse and often challenging conditions, such as outdoor recordings or instances with significant background noise. These factors can mask or distort speaker-specific cues, making it harder for the model to correctly identify the speaker.

In our examination of the depth video modality, we found that errors frequently stemmed from the low resolution of the RGB videos, which was particularly noticeable in the VoxCeleb2 dataset. This resulted in less accurate depth estimation which, in turn, affected the precision of the spatial representation of lip movements, subsequently lowering the identification performance. It is worth noting that the depth estimation algorithm is specifically trained on facial features and thus performs optimally with high-resolution RGB videos, as is the case with the KinectsDigits and TCD-TIMIT datasets. Therefore, when applied to lower-resolution videos, such as those in the VoxCeleb2 dataset, the depth estimation’s accuracy diminishes. The lower-resolution videos also struggle to capture the finer details of lip movements, limiting the depth modality’s discriminatory ability and potentially leading to identification errors.

## 5. Conclusions

This study demonstrates the effectiveness of a multimodal approach for speaker identification that incorporates both audio and depth information to achieve more accurate and reliable results. Through the examination of three benchmark datasets—KinectsDigits, VoxCeleb2, and TCD-TIMIT—we showed that the fusion of these modalities leads to significant improvements in identification performance. The proposed spatiotemporal architecture effectively extracts spatial and temporal features from depth information, while the x-vectors architecture processes the audio modality.

Our findings highlight the importance of integrating multiple modalities to overcome the limitations posed by short utterances and variable recording environments. By fusing audio and depth data, we achieved an enhanced performance in a range of scenarios, including clean recording environments and more realistic, constrained situations. The results indicate that depth information plays a crucial role in performance enhancement, with the addition of audio providing complementary benefits.

This research contributes to the advancement of speaker identification systems by proposing a robust multimodal approach. In our future work, we aim to propose an architecture based on early fusion, taking into account the high correlation between the lip movement and the voice generated. This would potentially lead to further improvements in performance and system reliability.

Future work could also extend this study by exploring the integration of other modalities or refining the fusion techniques to further improve the performance. Additionally, the application of the proposed methodology to other related tasks, such as speaker verification or emotion recognition, could provide valuable insights and contribute to the development of more advanced and reliable systems in these domains.

## Figures and Tables

**Table 1:** Data characteristics after word segmentation.

Dataset/Information	#spk	#avg/spk	(min, max, Mean)	fps	Mouth Crop $\left(h_{x},h_{y}\right)$
Kinects Digits [3]	30	10	(35 ms, 2.02 s, 600 ms)	30	(104,80)
TCD-TIMIT [30]	59	470	(35 ms, 2.07 s, 610 ms)	30	(300,150)
VoxCeleb2 [17]	1000	815	(40 ms, 1 s, 520 ms)	25	(60,60)

**Table 2:** Identification accuracy per view of the depth video.

*View/Dataset*	*XY*	*XT*	*TY*	*Spatio-Temporal Fusion*
KinectsDigits (Real Depth)	96.66%	91.66%	83.33%	**98.33%**
KinectsDigits (Estimated Depth)	**100%**	**100%**	**100%**	**100%**
TCD-TIMIT (Estimated Depth)	96.20%	83.33%	86.93%	**98.58%**
VoxCeleb2 (Estimated Depth)	11.82%	8.62%	8.58%	**17.49%**

**Table 3:** Identification accuracy per modality and their fusion for the three benchmarks.

*Modality/Dataset*	*Audio*	*Depth*	*Multimodal Fusion*
KinectsDigits (Real Depth)	75%	98.33%	**100%**
KinectsDigits (Estimated Depth)	75%	**100%**	**100%**
TCD-TIMIT (Estimated Depth)	89.25%	98.58%	**99.76%**
VoxCeleb2 (Estimated Depth)	56.03%	17.49%	**64.11%**

## Data Availability

Data available upon reasonable request.
